# Shortwave absorption by wildfire smoke dominated by dark brown carbon

**DOI:** 10.1038/s41561-023-01237-9

**Published:** 2023-08-07

**Authors:** Rajan K. Chakrabarty, Nishit J. Shetty, Arashdeep S. Thind, Payton Beeler, Benjamin J. Sumlin, Chenchong Zhang, Pai Liu, Juan C. Idrobo, Kouji Adachi, Nicholas L. Wagner, Joshua P. Schwarz, Adam Ahern, Arthur J. Sedlacek, Andrew Lambe, Conner Daube, Ming Lyu, Chao Liu, Scott Herndon, Timothy B. Onasch, Rohan Mishra

**Affiliations:** 1 Center for Aerosol Science and Engineering, Department of Energy, Environmental, and Chemical Engineering, Washington University in St Louis, St Louis, MO USA; 2 Institute of Materials Science and Engineering, Washington University in St Louis, St Louis, MO USA; 3 Department of Mechanical Engineering and Materials Science, Washington University in St Louis, St Louis, MO USA; 4 Center for Nanophase Materials Sciences, Oak Ridge National Laboratory, Oak Ridge, TN USA; 5 Department of Atmosphere, Ocean and Earth System Modeling Research, Meteorological Research Institute, Tsukuba, Japan; 6 Chemical Sciences Laboratory, NOAA Earth System Research Laboratories, Boulder, CO USA; 7 Cooperative Institute for Research in Environmental Sciences (CIRES), University of Colorado, Boulder, CO USA; 8 Environmental and Climate Sciences, Brookhaven National Laboratory, Upton, NY USA; 9 Aerodyne Research, Inc., Billerica, MA USA; 10 Department of Chemistry, University of Alberta, Edmonton, Alberta Canada; 11 China Meteorological Administration Aerosol–Cloud–Precipitation Key Laboratory, School of Atmospheric Physics, Nanjing University of Information Science and Technology, Nanjing, China; 12 Present Address: Institute of Chemical Physics, School of Chemistry and Chemical Engineering, Beijing Institute of Technology, Beijing, China; 13 Present Address: Department of Materials Science and Engineering, University of Washington, Seattle, WA USA; 14 Present Address: Ball Aerospace, Broomfield, CO USA

**Keywords:** Atmospheric science, Environmental impact, Climate sciences

## Abstract

Wildfires emit large amounts of black carbon and light-absorbing organic carbon, known as brown carbon, into the atmosphere. These particles perturb Earth’s radiation budget through absorption of incoming shortwave radiation. It is generally thought that brown carbon loses its absorptivity after emission in the atmosphere due to sunlight-driven photochemical bleaching. Consequently, the atmospheric warming effect exerted by brown carbon remains highly variable and poorly represented in climate models compared with that of the relatively nonreactive black carbon. Given that wildfires are predicted to increase globally in the coming decades, it is increasingly important to quantify these radiative impacts. Here we present measurements of ensemble-scale and particle-scale shortwave absorption in smoke plumes from wildfires in the western United States. We find that a type of dark brown carbon contributes three-quarters of the short visible light absorption and half of the long visible light absorption. This strongly absorbing organic aerosol species is water insoluble, resists daytime photobleaching and increases in absorptivity with night-time atmospheric processing. Our findings suggest that parameterizations of brown carbon in climate models need to be revised to improve the estimation of smoke aerosol radiative forcing and associated warming.

## Abstract (web summary)

Atmospheric short-wave absorption due to wildfire smoke is caused predominantly by dark brown carbon particles, according to observations from smoke plumes in the United States.

## Main

Wildfire smoke aerosols cause strong atmospheric warming and large surface cooling that is as important to Earth’s changing climate as carbon dioxide and other greenhouse gases^1,2^. The mass composition of smoke aerosols is mostly (>95%) organic (OA) with minority (<3%) fractions of inorganics and graphitic black carbon (BC)^2,3^, with the latter considered to be the dominant absorber of incoming shortwave solar radiation^4^. The light absorption characteristics of OA vary widely and remain poorly constrained in climate models^5,6^. Currently, atmospheric warming contributions from light-absorbing OA residing in a plume are either ignored or considered negligible owing to photobleaching compared with the relatively non-reactive BC in model parameterizations^2,7,8^.

The traditional view holds that chromophores of OA absorb predominantly at short wavelengths of visible light but negligibly at longer visible wavelengths, resulting in a brownish or yellowish visual appearance, hence, the optical name ‘brown carbon’ (BrC)^9,10^. The common techniques used to measure BrC in a laboratory or field setting involve solvent extraction of the soluble organic fraction of particulate matter followed by measurement of bulk absorbance using ultraviolet (UV)–visible–infrared spectrophotometry^11–13^. Measured absorbance of soluble BrC is subsequently converted to imaginary refractive index *k* that typically spans values between 10^−4^ and 10^−2^ across the wavelength *λ* range of 380 and 500 nm^6,14,15^. Thus, the soluble BrC component of smoke is weakly absorbing compared with BC, which has a high *k* ≈ 0.63 across the UV–visible–near-infrared spectra^16^. Moreover, BrC is highly susceptible to bleaching or loss of light absorbing ability within hours to days of emission^17–19^.

Recent laboratory studies^14,20–23^ indicate the presence of dark BrC components (d-BrC) in biomass-burning smoke that absorb strongly across the visible and near-infrared wavelengths. This class of BrC has low volatility, is insoluble and has high *k* values ≈ 0.2–0.4 in the visible spectrum^6,15^. The d-BrC component has been shown in laboratory burns to comprise 5–15% of smoke OA mass, with the remaining fraction composed of weakly absorbing, soluble BrC^14^. Observational evidence of d-BrC in wildfire smoke plumes and its significance with respect to atmospheric shortwave absorption remain elusive.

## Contribution of BC to absorption

We integrated bulk- and particle-scale observations to characterize the optical and physicochemical properties of the dominant light-absorbing components in the smoke plumes of western-US wildfires. This study was part of the 2019 NASA (National Aeronautics and Space Administration)/National Oceanic and Atmospheric Administration-sponsored Fire Influence on Regional to Global Environments and Air Quality field campaign^24^ to investigate the plume composition of western wildfires. A suite of aerosol and gas characterization instruments was operated aboard the ground-based Aerodyne Mobile Laboratory and NASA’s Douglas DC-8 aircraft. Synchronized measurements, to the extent possible, were conducted on each platform to intercept and study plumes during the 2019 wildfire season from near (less than 3 km) the fire management area through to the troposphere (10–11 km altitude).

**Fig. 1:**
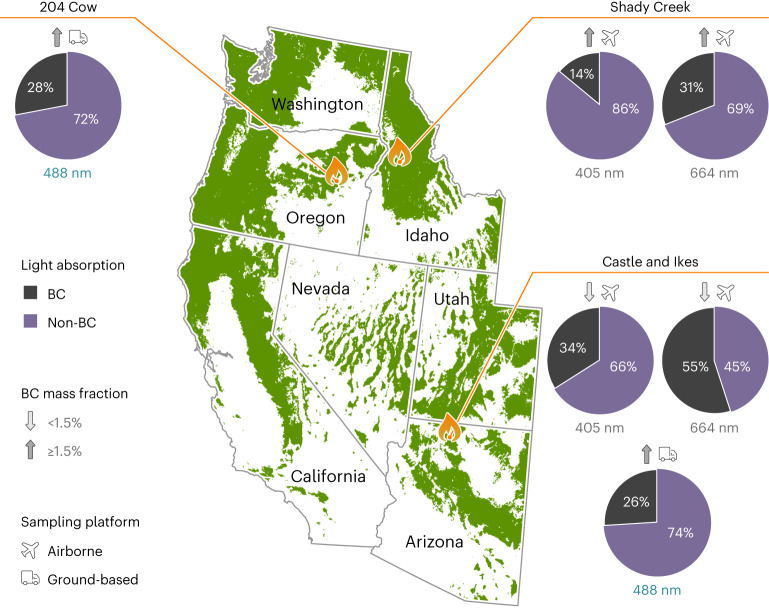
Shortwave absorption contributions by aerosols during the 2019 wildfire season in western United States. In situ ground and airborne measurements of refractory BC mass concentration and total aerosol light absorption by the SP2 and PAS, respectively, in smoke plumes of three wildfires—Shady Creek (Idaho), 204 Cow (Oregon) and Castle and Ikes (Arizona)—during July and August of 2019. The pie charts depict mean relative contributions by BC and non-BC components to total light absorption at wavelengths 405 nm and 664 nm (aircraft) and 488 nm (ground). Total mass fractions of refractory BC and non-refractory inorganic and organic components in aerosols near the fire emission sites are shown in Extended Data Fig. 1. Tree coverage data from radicalcartography.net courtesy of William Rankin.

**Extended Data Fig. 2:**
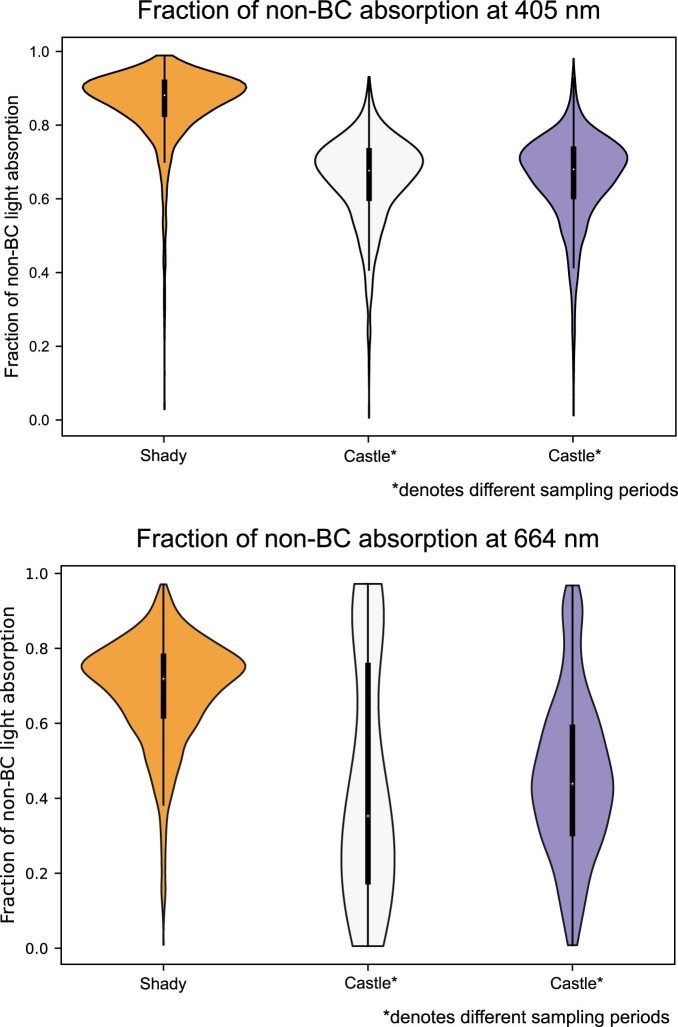
Fraction of non-BC light absorption in the troposphere. Violin plots showing the fraction of non-BC light absorption determined from tropospheric measurement datasets (n = 33 samples) of the SP2 and PAS onboard NASA’s DC-8 aircraft. An enhancement factor of 1.5 was included to account for black carbon ‘lensing effect’ in this calculation. At 405 nm, the non-BC light absorption contribution in the Shady and Castle fires were 0.86 ± 0.10 and 0.65 ± 0.12, respectively. At 664 nm, the non-BC light absorption contribution in the Shady and Castle fires were 0.7 ± 0.14 and 0.46 ± 0.25, respectively. Violin plots show the shape of a data set by using a Probability Density Function (PDF), or a density plot, which is effectively a smoothed-over histogram. The width of the PDF describes how frequently that value occurs in the data set. The wider regions of the density plot indicate values that occur more frequently. Violin plots include a boxplot that is used to show the minimum, first quartile, median, third quartile, and maximum.

First-principles instruments, two multiwavelength photoacoustic spectrometers (PAS)^25^ and two single-particle soot photometers (SP2)^26^, measured the in situ bulk aerosol light absorption coefficients and the refractory BC mass concentrations, respectively. Figure 1 summarizes the PAS and SP2 results of sub-micrometre smoke aerosol from the Shady Creek (Idaho), Castle and Ikes (Arizona) and 204 Cow (Oregon) fires, sampled between July and August 2019. Light absorption by BC alone (less than 3% of total aerosol mass) could not account for the total sub-micrometre aerosol absorption. Non-BC light-absorbing aerosol components contributed roughly three-fourths and half of the total absorption in the blue (405/488 nm) and red (664 nm) wavelengths, respectively (see Extended Data Fig. 2 for additional analysis). The contribution of the non-BC light-absorbing component to the total absorption increased with increasing BC mass fractions in the plume. This trend provided an inkling of d-BrC as the dominant non-BC absorbing component of smoke plumes^15,21^. This is further corroborated by the observation of negligible absorption contribution at 664 nm by the water-soluble BrC component of smoke^27^.

## Abundance of d-BrC tar balls

**Fig. 2:**
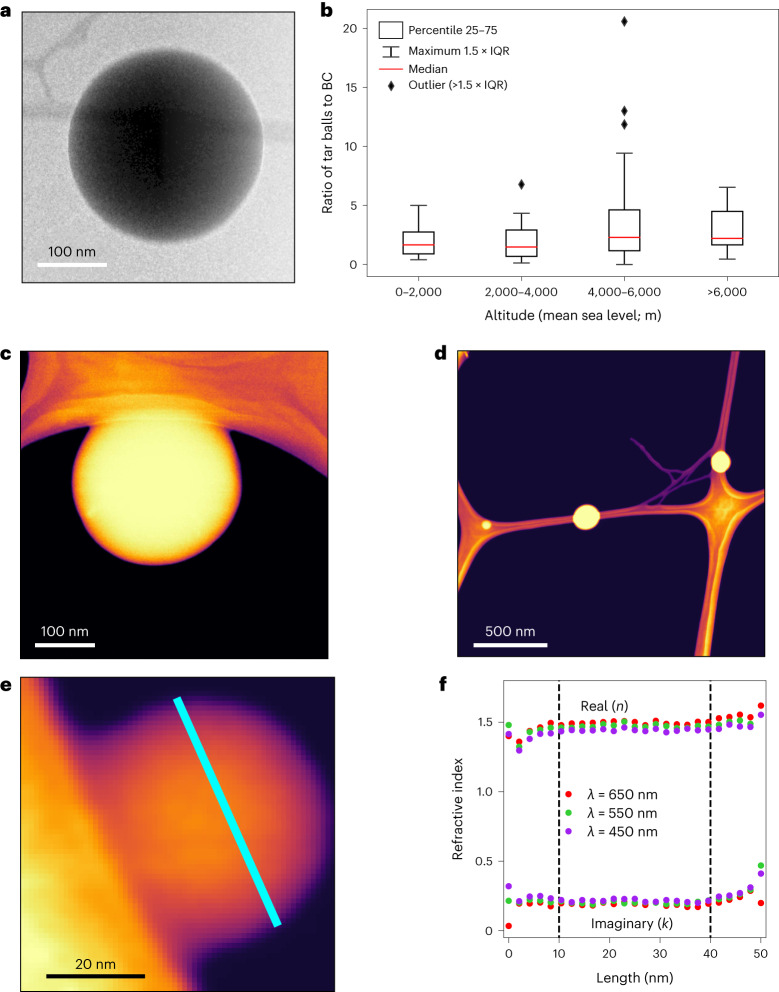
d-BrC tar balls abundant in smoke plumes. **a**, TEM image of a d-BrC tar ball abundant in the smoke plumes sampled at altitudes ranging from ground to 10 km. Identification of these tar balls involves use of secondary electron imaging at low accelerating voltage and low working distance^29^. **b**, Relative abundance of d-BrC tar balls and BC as a function of sampling altitude along the smoke plume height. The total number of particles analysed was *n* = 3,837. **c**, High-angle annular dark field (HAADF) image^48^ of a single d-BrC tar ball with a diameter of ~290 nm sampled close to a fire. **d**, HAADF images of multiple d-BrC tar balls with diameters of 70 nm, 150 nm and 185 nm. A Nion HERMES scanning TEM^28^ was used for acquiring these images. **e**, HAADF image of a tar ball with a diameter of ~50 nm, acquired simultaneously with EEL spectra. **f**, Variation in the real (*n*) and imaginary (*k*) refractive index across the diameter of the tar ball. The data points corresponding to the diameter of the tar ball are highlighted in **e** with the cyan-coloured line. The *n* and *k* values, corresponding to wavelengths of *λ* = 450 nm, 550 nm and 650 nm, remained consistent for all the three wavelengths for EEL spectra collected >10 nm from the particle edges. The particles show a high degree of material homogeneity and uniformity in refractive index across their physical cross sections.

To validate our conjecture of d-BrC’s probably significant contribution to absorption, we employed transmission electron microscopy (TEM) and low-loss electron energy-loss spectroscopy (EELS)^28^ analysis at the single-particle scale. This analysis facilitated the identification and detailed physicochemical and optical characterization of extremely low-volatility organic fractions of smoke that survive in the vacuum environment of an electron microscope. Our statistical analysis of around 4,000 particles from all the sampled wildfire episodes found an abundance of viscous and low-volatility tar balls^29^ (Fig. 2a,c,d, Supplementary Figs. 2 and 3 and Supplementary Table 1). ‘Tar balls’ is a term used communally to refer to the thermally stable morphology of viscous spherical atmospheric OA^30^. These spherical particles are a subset of BrC^6,31,32^ and have been shown to exhibit a continuum of optical properties. Their *k* values could span orders of magnitude between 10^−3^ and 10^−1^ across the short visible wavelengths^6,33–36^.

For energy-loss values between 1.0 and 4.1 eV, monochromatic low-loss EELS facilitates an accurate determination of a particle’s dielectric function and, thereby, its refractive index in the *λ* range of 300 to 1,200 nm. Figure 2f shows the variation in the real (*n*) and imaginary (*k*) refractive index, corresponding to wavelengths *λ* = 450, 550 and 650 nm, measured across the diameter of a typical 50 nm tar ball (Fig. 2e). The high *k* values for all the three wavelengths confirmed that the tar balls belong to the category of d-BrC. The particles demonstrated remarkable thermal stability in composition and morphology when heated to a temperature of 160 °C in vacuum, which corresponds to 465 °C at atmospheric pressure.

The relative abundance of d-BrC tar balls in the plumes was four times greater than BC (Supplementary Table 2). This ratio of 4/1 remained approximately constant with increasing altitude from ground up to 10 km (Fig. 2b). The mean area-equivalent sphere diameters of the particles ranged from 140 to 200 nm with a geometric standard deviation between 1.4 and 1.6. They comprised between 5% and 26% of the total aerosol mass concentration in plumes.

**Fig. 3:**
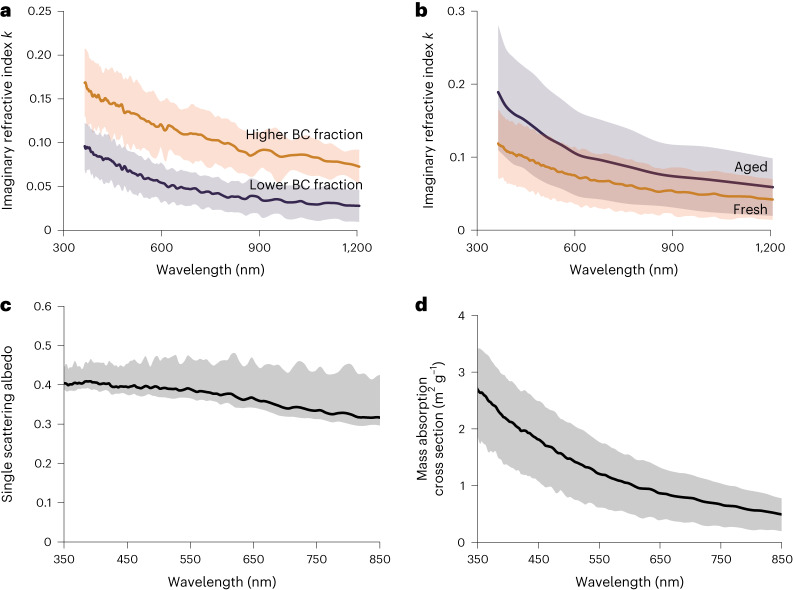
Spectral optical properties of d-BrC. **a**, Mean imaginary part (*k*) of the complex refractive index, derived from EEL spectra, against optical wavelength *λ* for the analysed d-BrC tar balls corresponding to the three wildfires. The *k* values showed sensitivity to co-emitted BC mass fractions in the smoke plumes. **b**, Enhancement in the spectral *k* values on atmospheric ageing, dictated predominantly by night-time NO_3_· oxidation. The particles showed resistivity to daytime OH· oxidation over three equivalent days (Extended Data Fig. 6). The shaded regions in the plots represent errors corresponding to one standard deviation of the measurements. Power-law scaling coefficients with mean and error bars (one standard deviation) for the measured *k* values have been tabulated in Extended Data Table 1. **c**,**d**, Single scattering albedo (**c**) and mass absorption cross sections of the particles (**d**). Shaded region corresponds to one standard deviation and accounts for uncertainties in density, particle size distribution, refractive index and individual measurements.

**Extended Data Fig. 3:**
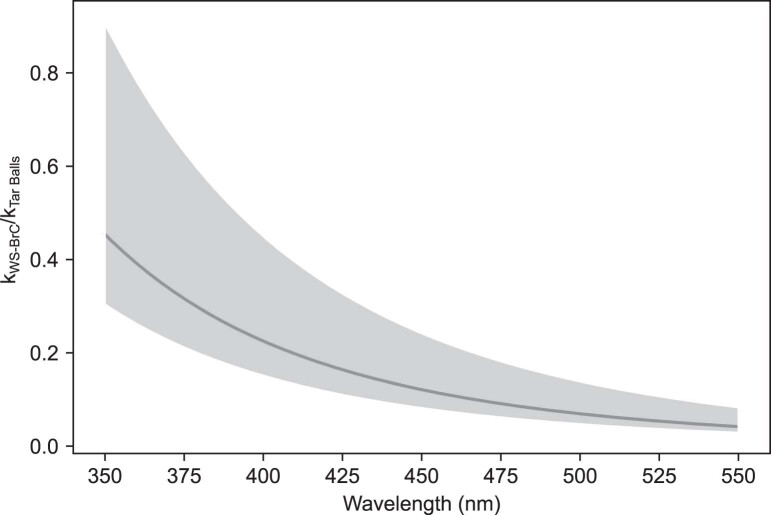
Imaginary refractive index of water-soluble and dark BrC. Ratio of spectral imaginary refractive index of water-soluble BrC (k_WS-BrC_) and d-BrC tar balls observed during this study. Water-soluble BrC refractive index was measured using spectrally-resolved light absorption in water extracts of particles from fresh smoke collected on filters at ground level. Water extraction of aerosol particles collected on filters from wildfire smoke typically accounts for approximately half of the overall solvent extractible BrC species^58^. The shaded region in the plot represent errors corresponding to one standard deviation of the measurements.

**Extended Data Fig. 4:**
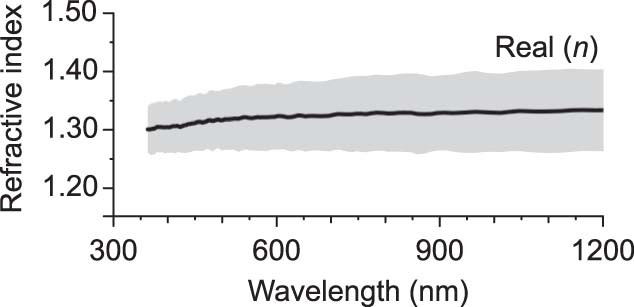
Real refractive index of dark BrC. Spectrally resolved real (n) = 1.31 ± 0.03 of the complex refractive index values of d-BrC tar balls sampled across all fires in this study. The shaded region represents errors corresponding to one standard deviation of the measurements.

Figure 3a shows the mean imaginary refractive component *k* of all EELS-analysed d-BrC tar balls against *λ*. Power-law scaling relations of the form *Y* = *Y*_0_*S*^*β*^, where *Y* is *k*, *S* is *λ*, *Y*_0_ is the prefactor and *β* is the power-law exponent, emerge for the wavelength-dependent *k* values (Extended Data Table 1). We observe *k* values to decrease in *λ*^−2/3^ and *λ*^−1^ power laws depending on high (>1.5%) and low (<1.5%) BC mass fractions, respectively. By comparison, soluble BrC measurement in water extracts of particle-laden filters collected on ground yield order-of-magnitude lower *k* values (Extended Data Fig. 3). The real part *n* stayed wavelength invariant at 1.31 ± 0.03 (Extended Data Fig. 4). This value is consistent with previous measurement of *n* from wildfire smoke^37^.

**Extended Data Fig. 5:**
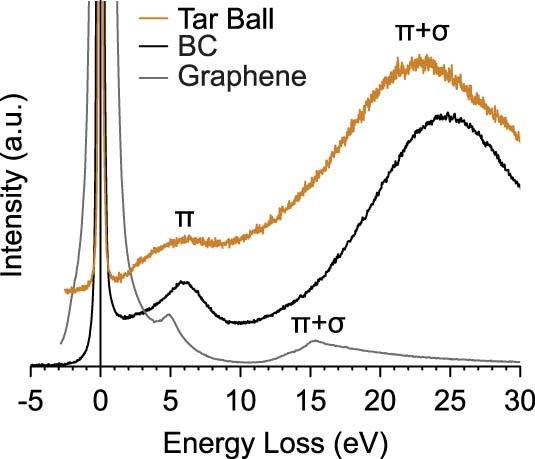
Orbital Hybridization. The low-loss EEL spectra < 30 eV of a typical d-BrC tar ball in comparison to those of a co-emitted BC aggregate and a reference single-layer graphene sample^59^. The π peak, which represents the π electron excitation, is observed to be at ~5 eV for graphene and ~6 eV for both carbonaceous aerosol types. The broadening of the π peak as we go from single-layer graphene to the observed tar ball is a measure of increasing amorphization, or decreasing graphitization. This is associated with decreasing sp^2^ hybridization of neighboring carbon atoms, and hence relative lowering of π- π transitions^53^. The tar ball is much less graphitized than the carbon in BC, in accordance with its lower sp^2^ hybridization. This points to its formation along the BC formation pathway in high-temperature flames. The formation process of tar balls likely includes carbonization but not graphitization, and therefore, they do not convert to the thermodynamically favorable allotrope of graphitic carbon upon heat treatment in the fire^32^. The π+σ peak, which represents the excitations involving all valence electrons, is observed at ~15 eV for graphene and ~22–25 eV for tar ball and BC. It has been previously shown that the π+σ peak for graphene shifts to higher energies as the number of layers increase, and a third broad peak appears at around 25 eV^59^. This broad peak at ~25 eV is a characteristic feature of graphitic samples.

There remains considerable ambiguity in the formation mechanisms of tar balls in combustion systems^30,32^. In this study, we hypothesize that the d-BrC tar balls are formed as primary particles via heat-induced carbonization^32^ along the BC formation pathway in the high-temperature flaming zones of a wildfire. Comparative analysis of chemical bonding characteristic between tar balls and BC samples collected close to the fires strengthens this hypothesis (Extended Data Fig. 5).

We observed that the *k* values and the wavelength dependence of d-BrC depended strongly on mass fractions of co-emitted BC and burn conditions. For example, the *k* values at 550 nm, corresponding to a high-temperature flaming phase of the Shady Creek Fire containing high BC fractions, were 0.13 ± 0.04. When the fire transitioned to a mixed phase, dominated by a low-temperature smouldering phase with relatively low BC mass concentrations, the *k* values decreased to 0.06 ± 0.03. It is likely that with the lowering of flame temperature, the degree of graphitization of carbon atoms^38^ in tar balls decreases, resulting in low *k* values.

## Effects of atmospheric ageing on d-BrC

During measurements of both the Castle and Ikes and the 204 Cow fires, the ground-based team frequently encountered episodes wherein the plumes contained negligible (<0.5%) or below-detection-limit refractory BC mass fractions (Supplementary Fig. 1). Smoke aerosol absorption corresponding to such episodes was almost entirely tar ball dominated. Corresponding to these episodes, the smoke plumes were oxidized using OH· and NO_3_· radicals in a photochemical reactor^39^ to mimic daytime and night-time oxidation, respectively. In the reactor, we artificially increased the oxidant concentration to various levels to achieve ‘equivalent time’ exposures of longer than 72 h. We use the concept of equivalent time, which is the product of oxidation concentration and actual reactor residence time, which equates to a real-world span of time when exposed to the average oxidant concentration present in the real atmosphere (see Supplementary Text for details).

Over three equivalent days and nights (approximately 84 equivalent hours) of atmospheric ageing, we measured the relative change in absorption for tar balls downstream of our reactor using our PAS instrument. We quantified the change in absorption by comparing measurements of their absorption coefficients when OH· or NO_3_· was present with those when oxidants were absent. We observed that NO_3_· oxidation leads to an enhancement in particle light absorption coefficients by a factor of 1.5 ± 0.1 (ref. ^39^). Using this factor, we calculated the corresponding enhancement in tar balls’ *k* values (Fig. 3b). Enhancement in absorption from night-time oxidation is probably from secondary reactions of organics involving addition of nitrogen-containing functional groups (Supplementary Fig. 4), which in turn could act as additional chromophores for particle light absorption^40^.

**Extended Data Fig. 6:**
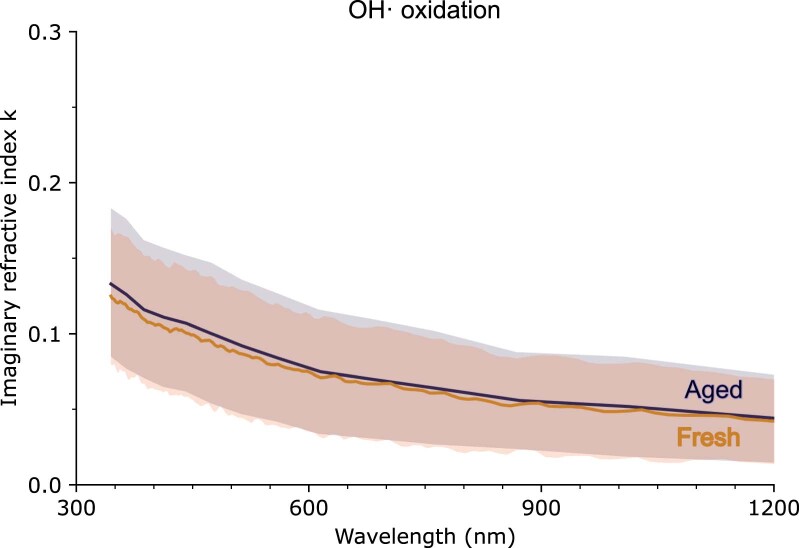
Daytime oxidation effects on imaginary refractive index. The spectral *k* values upon daytime OH· oxidation over a duration of three equivalent days. The slight fluctuations in *k* values of the aged plume is likely due to gas-phase oxidation of volatile organic compounds in the plume, leading to formation of secondary BrC^60^. The shaded regions in the plots represent errors corresponding to one standard deviation of the measurements.

Daytime OH· oxidation for the same duration showed a net-zero change in absorption coefficients and, by extension, negligible change in *k* values (Extended Data Fig. 6). This confirms that d-BrC tar balls resist photochemical bleaching over an extended period in the atmosphere. The rate of bleaching has been shown to slow substantially with increasing viscosity of aerosols^41^. In the case of tar balls, we hypothesize that their high viscosity limits the surface and bulk reaction rates, as well as the diffusion rates between chromophores and oxidants within the particle.

## Radiative properties and implications of d-BrC

**Extended Data Fig. 7:**
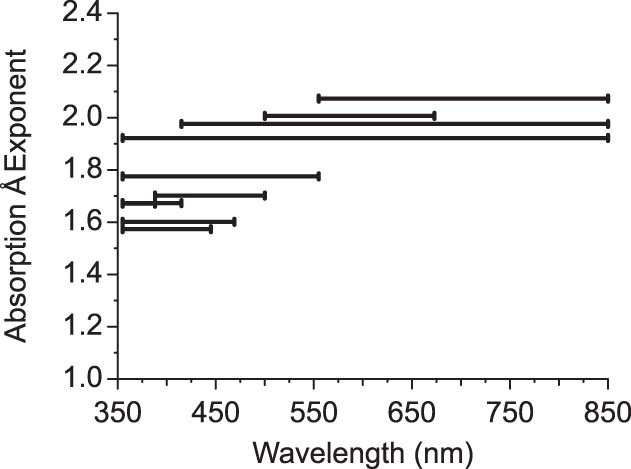
Absorption Ångström Exponent of dark BrC. Absorption Ångström Exponent (AÅE) across the near-UV and visible spectra in wavelength intervals corresponding to different wavelength intervals used extensively for detecting and characterizing wildfire smoke plumes^45,61^. The two ends of a solid black line represent the two wavelengths *λ*_1_ and *λ*_2_ between which the AÅE, which is defined as the exponent in a power law expressing the ratio of the mean absorption coefficients (*λ*_1_, *λ*_2_), is calculated. A total of 33 smoke samples were analyzed for the calculation of AÅE. Only one estimate of AÅE is available, so the standard error cannot be calculated directly.

Using the spectral refractive indices from EELS and the TEM size distribution measurements, we determined the particle mass absorption cross section (MAC)^4^ and single scattering albedo (SSA, the ratio of scattering and extinction efficiencies)^9^ (Fig. 3c,d). Climate models rely on the accuracy of these optical parameters to estimate the influence of atmospheric aerosols on Earth’s radiation balance^4,42^. The SSA is tightly constrained at 0.38 ± 0.03 across the visible spectrum. We calculated a mean MAC of ~3.0 m^2^ g^–1^ at *λ* = 350 nm that decreases slowly in *λ*^−1.8^ power-law behaviour in the visible spectrum. The particle absorption Ångström exponents (AÅEs, the rate at which absorption cross sections vary with *λ*) range between 1.5 and 2.0 across different *λ* intervals in the near-UV–visible spectrum (Extended Data Fig. 7).

The magnitude and wavelength dependencies of SSA and *k* (Fig. 3a–c) of d-BrC tar balls render these particles distinctly indistinguishable from BC in the atmosphere^4^. Absorption contributions from d-BrC have remained unaccounted for in previous field studies of biomass burning that used exclusively solvent extraction techniques for probing BrC characteristics^11–13,27^. The observed abundance of d-BrC tar balls in this study should provoke a rethinking of OA’s role in shortwave radiative forcing because it may significantly enhance current model predictions of atmospheric warming by BrC^7,8,41^.

Our findings are particularly timely given that aerosol emissions from wildfire events are increasing in magnitude across the western United States and rest of the world due to climate change caused by anthropogenic activities^43^. Our findings can be used to better constrain the radiative forcing estimates of BrC aerosols in climate models^5^, as well as improve satellite- and ground-based retrievals of wildfire smoke^44,45^. Future research is necessary to better understand the aerosol–climate impacts of these particles^46,47^.

## Methods

Details regarding the wildfire episodes, sampling strategy and instrumentation can be found in the Supplementary Information.

### Estimation of non-BC light absorption

The total aerosol light absorption was measured using PASs that were deployed aboard the NASA DC-8 aircraft and the Aerodyne Mobile Laboratory (AML). The PAS on the DC-8 measured dry aerosol absorption coefficients of particles at wavelengths of 405, 532 and 664 nm, while the PAS (also called the MIPN) aboard the AML collected absorption data at 488 and 561 nm. An SP2 aboard both platforms measured the refractory black carbon (rBC) mass concentration. All the instruments recorded data at a frequency of 1 Hz, except for the PAS aboard the AML, which recorded data every 2 seconds.

The PAS data with absorption coefficient (*b*_abs_) values < 5 M m^−1^ and SP2 measurements with rBC mass concentration values < 50 ng standard (std) m^−3^ were removed from our calculations to reduce noise in the data. The product of the rBC mass concentration from the SP2 and a BC mass absorption coefficient (MAC_BC_) of 11.25 m^2^ g^−1^, after accounting for a mean absorption enhancement factor of 1.5 for aged BC due to the presence of organic coatings, was used to estimate the BC light absorption coefficient (*b*_abs,BC_) at 550 nm (ref. ^49^). The value of *b*_abs,BC_ at 550 nm was extrapolated to obtain the *b*_abs,BC_ values at the 405 and 488 nm using an AÅE value of 1 (ref. ^50^) using:

1 $${b}_{{{\mathrm{abs}}},{{\mathrm{BC}}}}\left(\lambda \right)={b}_{{{\mathrm{abs}}},{{\mathrm{BC}}}}(550)\times{\left(\frac{\lambda }{550}\right)}^{-{\mathrm{A}}{{\text{\AA}} \rm{E}}}$$

where *λ* is the wavelength at which the absorption coefficient needs to be estimated. It is noteworthy that the distribution of BC among diverse particles of varied composition, as well as the assumed value for MAC_BC_ and the fixed absorption enhancement factor, could give rise to deviation in the assumed mean AÅE value of 1 (refs. ^49,51^). The light absorption coefficient by the non-BC component at a particular wavelength was estimated as the difference between the *b*_abs_ and the *b*_abs,BC_ values.

### Sampling and measurements of particles using TEM

#### Airborne

Smoke aerosol particles were sampled using an impactor sampler (AS-24W, Arios Inc.) with TEM grids with Formvar substrates (U1007, EM-Japan). The 50% cut-off sizes used for the TEM grid sampling were 100 nm and 700 nm, respectively, in aerodynamic diameters. Sampling was conducted to cover each transect of smoke, with sampling times of ~1 to 3 min and an airflow rate of 1.0 l min^–1^. For this study, 9 TEM grids (3,275 particles in total) were analysed corresponding to samples from the Castle and Ikes and Shady Creek fires using a scanning TEM (STEM; JEM-1400, JEOL) equipped with an energy-dispersive X-ray spectrometer (EDS; X-Max 80 mm, Oxford instruments). An acceleration voltage of 120 keV and an acquisition time of 20 s was used for STEM-EDS measurements. We chose two or three areas with ~100 particles at a magnification of 6000× in STEM mode. Volatile and semi-volatile aerosol particles such as volatile organic compounds and nitrates would be lost after the sampling and in the vacuum TEM chamber. The smallest particle cut-off size for STEM-EDS analyses was an area-equivalent diameter of 0.25 µm in the STEM images. Relative weight percentages within each particle measured by STEM-EDS were obtained for C, N, O, Na, Mg, Al, Si, P, S, Cl, K, Ca, Ti, Mn, Fe and Zn. The size-dependent number fractions are shown in Supplementary Fig. 3. Carbonaceous particles dominate in most particle sizes.

In addition to their composition, we classified the measured particles on the basis of their electronic darkness or brightness and sphericity in the TEM images (Supplementary Fig. 2). ‘Electronically dark’ particle^29^ is defined for a particle mean intensity less than 70, where a black pixel has the intensity of 0 and a white pixel has the intensity of 255 within all particles. Darker particles in TEM images indicate that they transmit less electron beam by absorbing or scattering it. These darker particles are thicker than other particles (tilted TEM image of Supplementary Fig. 2). The darker particles are the d-BrC tar balls, and they appear thick and highly viscous on the TEM grids due to low or negligible deformations during the impactor sampling. Tilted TEM images show the particle thickness and support the assumption that the darker particles are thicker compared with other particles. The number fractions are shown in Supplementary Table 1.

#### Ground

An MP-3 microanalysis particle sampler (California Measurements, Inc.) on board the AML was used to sample smoke particles from the fires. The particles were deposited onto Cu TEM grids coated with lacey-carbon support films. Sampling duration was about 1–2 min at a volume flow rate of 2 lpm. We collected around 43 TEM grids of smoke aerosols. Several hundred particles were imaged using a scanning TEM (FEI Tecnai G2 Spirit) equipped with a STEM-EDS and an aberration-corrected and monochromated Nion HERMES scanning TEM at Oak Ridge National Laboratory. This analysis confirmed the overwhelming presence of electronically dark tar balls^29^ in fresh smoke samples.

Thirty-three samples were analysed for EELS data at Oak Ridge National Laboratory using a Nion spectrometer equipped with a Hamamatsu ORCA SCMOS detector. The microscope was operated at an accelerating voltage of 60 kV with a probe convergence of 30 mrad. The collection angle for the EEL spectrometer was 25 mrad with a dispersion of 20 meV channel^–1^. The EELS datasets were acquired as spectrum images with a dwell time of about 50 ms pixel^–1^ and total acquisition time of ~5 min for each dataset, which contained approximately 70 × 70 pixels (pixel size about 1–3 nm). The zero-loss peak (ZLP) obtained after monochromation for all EELS datasets had a full width at half maximum of about 100–150 meV. To remove surface contaminants, the TEM grids were heated at 160 °C for about 6 h under vacuum before STEM–EELS experiments, which corresponds to an evaporation temperature of approximately 465 °C at atmospheric pressure. The evaporation temperature was estimated using the Clausius–Clapeyron equation^52^.

### EELS

We used the following procedure to extract the optical properties of the aerosol particles from the low-loss EEL spectra. The first part of the analysis involves the removal of the ZLP, including its tail, from the EEL spectra. The ZLP was removed from the low-loss EEL spectra of the aerosol particles using a reflected-tail method, which has been shown to give reliable results for similar carbonaceous aerosol particles^53^. Supplementary Fig. 6 shows that the reflected-tail method is robust and does not introduce any inaccuracies in the retrieved dielectric constants and refractive indices in the wavelength range of interest.

We used Fourier-log deconvolution to remove plural scattering and obtain the single scattering distribution (*S*(*E*)), as a function of the energy loss *E*. Neglecting retardation effects and instrumental broadening, the *S*(*E*) can be expressed as a function of permittivity *ɛ*(*Ε*)^53,54^:

2 $$S(E\,)=\frac{{I}_{0}t}{\uppi {a}_{0}{m}_{0}{\nu }^{2}}\text{Im}\left[\frac{-1}{\varepsilon (E\,)}\right]\mathrm{ln}\left[1+{\left(\frac{\beta }{{\theta }_{E}}\right)}^{2}\right]+{S}_{S}(E\,),$$

where *I*_0_ is the intensity of the ZLP, *t* is the sample thickness, *a*_0_ is the Bohr radius, *m*_0_ is the rest mass of an electron, *ν* is the speed of the electron beam, Im[*f*] denotes the imaginary component of the function *f*, *β* is the collection angle and *θ*_*E*_ is the characteristic angle as a function of energy loss, given by ${\theta }_{E}=E/\gamma {m}_{0}{\nu }^{2}$. The factor *γ* = *m*/*m*_0_ takes account of the relativistic increase in mass of the incident electron. *S*_S_$(E)$, which describes the surface loss function, can be expressed with equation (3), assuming there is no coupling between the surfaces.

3 $${S}_{{\mathrm{s}}}(E\,)=\frac{{I}_{0}}{\uppi {a}_{0}{k}_{0}T}\left[\frac{{\tan }^{-1}\left(\,\beta /{\theta }_{E}\right)}{{\theta }_{E}}-\frac{\beta }{\left(\,{\beta }^{2}+{\theta }_{E}^{2}\right)}\right]\left[\frac{4{\varepsilon }_{2}}{{\left({\varepsilon }_{1}+1\right)}^{2}+{\varepsilon }_{2}^{2}}-\text{Im}\left(\frac{-1}{\varepsilon }\right)\right]$$

In equation (3), *k*_0_ is the wavenumber of the radiation, and *T* is *m*_0_*ν*^2^/2; *ε*_1_ and *ε*_2_ are the real and imaginary components of the dielectric function, respectively. To calculate the dielectric function from *S*(*E*), Kramers–Kronig analysis (KKA) was performed using HyperSpy^55^, which is an open-source Python software package for multi-dimensional data analysis. Specifically, for the KKA, we used the fast-Fourier transform method as described by ref. ^54^ within the thin-film approximation. The thickness of the film is estimated to be the diameter of the aerosol particles^53,54^. KKA works within the thin-film approximation and fails for EEL spectra collected close to the edges of the particle because of increased contribution from surface plasmons. In general, this approach for extracting the dielectric function from the low-loss EELS should be valid for particles larger than 50 nm provided the spectra are collected at least 10 nm away from the edges. For smaller particles of sizes between 20 nm and 50 nm, the refractive index can still be calculated from the low-loss EEL spectra collected close to the centre. The EELS data in this study were collected from the centres of the particles regardless of their size.

The Kramers–Kronig transformation is given by equation (4), where the real part of the dielectric function, Re[1/*ɛ*(*Ε*)], can be calculated from the imaginary part of the energy-loss function, Im[–1/*ɛ*(*Ε*)]. Here *Eʹ* corresponds to the energy losses, and *P* denotes the Cauchy principal value. The dielectric function can then be determined using equation (5). KKA is an iterative method where *S*_S_(*E*) is estimated at every step until the dielectric function converges.

4 $$\mathrm{Re}\left[\frac{1}{\varepsilon (E\,)}\right]=1-\frac{2}{\pi }P{\int }_{0}^{\infty }\text{Im}\left[\frac{-1}{\varepsilon (E^{\prime} )}\right]\frac{E^{\prime} {\mathrm{d}}E^{\prime} }{E^{{\prime}2}-{E}^{2}}$$

5 $$\varepsilon (E)={\varepsilon }_{1}(E\,)+i{\varepsilon }_{2}(E\,)=\frac{\mathrm{Re}\left[1/\varepsilon (E\,)\right]+i\text{Im}\left[-1/\varepsilon (E\,)\right]}{{\left\{\mathrm{Re}\left[1/\varepsilon (E\,)\right]\right\}}^{2}+{\left\{\text{Im}\left[-1/\varepsilon (E\,)\right]\right\}}^{2}}$$

Upon the determination of the dielectric function, the complex refractive index (*n* + *ik*) can be calculated using equations (6) and (7), where *n* and *k* are the real and imaginary parts of the refractive index, respectively, and *i* is the imaginary unit, which denotes the imaginary part of a function.

6 $$n={\left\{\frac{{\left[{{\varepsilon }_{1}}^{2}+{{\varepsilon }_{2}}^{2}\right]}^{1/2}+{\varepsilon }_{1}}{2}\right\}}^{1/2}$$

7 $$k={\left\{\frac{{\left[{{\varepsilon }_{1}}^{2}+{{\varepsilon }_{2}}^{2}\right]}^{1/2}-{\varepsilon }_{1}}{2}\right\}}^{1/2}$$

We validated the accuracy of our refractive index retrieval technique by applying it on BC sampled during the campaign (Supplementary Fig. 7). Our measurement values were in close agreement with those used in climate models^16^.

### Water-soluble BrC

Two 1/4 inch diameter punches were taken from the 47 mm quartz-fibre filter samples for solvent extraction. The particle-laden filter punches were placed in amber vials along with 800 µl water and sonicated for 1 h. Solvent extracts were then passed through Teflon membrane syringe filters (polytetrafluoroethylene, 0.22 µm, Fisherbrand) to remove any suspended insoluble particles introduced during the extraction process. The spectral light absorbance (*A*(*λ*)) of the solvent-extracted organics (or BrC) was measured using a UV–visible spectrophotometer (LAMBDA 35, PerkinElmer) at wavelengths ranging from 350 nm to 800 nm with a resolution of 1 nm. The complex part of the BrC refractive index was determined using:

8 $$\begin{array}{c}k\left(\lambda \right)=\frac{\rho \lambda \left(\frac{\alpha \left(\lambda \right)}{\rho }\right)}{4\uppi }\end{array}$$

where *k*(*λ*) is the imaginary part of the complex refractive index at wavelength *λ*, *ρ* is the density of organics in the solvent (1.4 g c^–3^) and *α*(*λ*)/*ρ* is the mass absorption efficiency, calculated as the ratio of the absorption coefficient of the organics in the solution (*b*_abs,sol_(*λ*)) to the mass concentration of dissolved organics. We used the absorbance value to calculate *b*_abs,sol_(*λ*) using the equation:

9 $${b}_{{{\mathrm{abs}}},{{\mathrm{sol}}}}\left(\lambda \right)=\left(A\left(\lambda \right)-A\left(700\right)\right)\frac{{V}_{l}}{{V}_{a}\times l}\times\,{\rm{ln}}(10)$$

where *A*(*λ*) is absorbance at wavelength *λ*, *V*_l_ is the volume of solvent used for extraction, *V*_a_ is the volume of air sampled through the filter punch area and *l* is the optical path length travelled by the beam (1 cm). The logarithm term is used to convert the base 10 logarithm from the absorbance measurements to a natural logarithm. The absorbance is normalized to measurements at 700 nm, *A*(700), to account for signal drift within the instrument.

### Change in optical properties due to photochemical ageing

The experimental set-up and details of the ageing experiments can be found in ref. ^39^. Wildfire smoke with d-BrC tar balls as the dominant light-absorbing component was sampled at two locations—Arizona and Oregon—and was oxidized using OH radicals to mimic daytime oxidation and NO_3_ radicals to mimic night-time oxidation. For the OH· oxidation experiments, we observed little to no change in MACs of d-BrC tar balls at 561 nm within ±2% over 3 equivalent days while the MAC increased by a factor of 1.47 ± 0.01 for the NO_3_· oxidation experiments over 3 equivalent nights. The refractive index (RI) calculations for the aged samples in Fig. 3b are based on results from the NO_3_· oxidation experiments.

The RI values for the aged samples were calculated using a Mie theory-based RI retrieval algorithm called PyMieScatt^56^. PyMieScatt outputs the aerosol RI using aerosol absorption and scattering coefficients at a particular wavelength of light, as well as the particle size distribution, as inputs. We used TEM images to determine the range of particle sizes in our sample and calculated the geometric mean diameter as well as the geometric standard deviation of a lognormal distribution that best fit the maximum size range obtained from TEM analysis. We observed organic aerosols with sizes ranging from 40 nm to 300 nm. The absorption coefficients for the ‘fresh’ emissions (*b*_abs,fresh_) were calculated using RI values from the EELS analysis and the lognormal size distribution obtained from the TEM analysis. The absorption coefficients for the ‘aged’ emissions (*b*_abs,aged_) were estimated by multiplying *b*_abs,fresh_ with an average enhancement factor of 1.47 as determined from the NO_3_· oxidation experiments for 3 equivalent nights of ageing. The scattering coefficients (*b*_scat_) for the fresh and aged particulate emissions, as determined by our multi-wavelength nephelometry measurements (Supplementary Information), were kept unchanged. The RI for the aged emissions was then calculated using PyMieScatt with *b*_abs,aged_, *b*_scat_ and the estimated lognormal size distribution as inputs.

The inverse calculations were computationally intensive; hence, only a subset (one in ten) of the detailed spectrum of fresh *k* values was used for our calculations of the *k*_aged_ spectrum (Fig. 3b). This led to a loss of the finer features in the *k*_aged_ spectrum compared with the *k*_fresh_ spectrum in Fig. 3b, but the method still retains the trends we expect for *k*_aged_.

### MAC, SSA and AÅE

We calculated the MACs of the d-BrC tar ball particles by dividing *b*_abs,fresh_ with the mass concentration of the tar balls—estimated using the lognormal size distribution obtained from the TEM analysis and a density of 1.6 g cm^–3^ (ref. ^34^). The AAE is defined for a pair of wavelengths *λ*_*1*_ and *λ*_*2*_ as the exponent in a power law expressing the ratio of the absorption coefficients *b*_abs_ (*λ*_1_, *λ*_2_) as^57^:

10 $${{\mathrm{AAE}}}\left({\lambda }_{1}{\lambda }_{2}\right)=\frac{\mathrm{ln}\left[{b}_{{{\mathrm{abs}}}}\left({\lambda }_{1}\right)/{b}_{{{\mathrm{abs}}}}\left({\lambda }_{2}\right)\right]}{\mathrm{ln}\left[{\lambda }_{2}/{\lambda }_{1}\right]}$$

Aerosol SSA is defined as the ratio of aerosol scattering coefficient *b*_sca_ and extinction coefficient *b*_ext_, which is the sum of *b*_abs_ and scattering coefficients. SSAs of the d-BrC tar balls were calculated by solving the Mie equations using the complex RI data obtained using EELS analysis and the lognormal size distribution obtained from the TEM analysis.

### Tar ball mass fraction

We estimated the mass concentration of the d-BrC tar balls in the sampled plumes. Cavity ring-down spectroscopy extinction data (for example, Supplementary Fig. 5) indicate that refractory particulate matter (PM; components observed on the TEM grids) comprises 10–50% of the total PM. Supplementary Tables 1 and 2 indicate that >85% by number (which corresponds to >88% by mass) of the total PM corresponds to organic aerosol particles. The d-BrC tar balls correspond to 0.58 ± 0.06 fraction of the total refractory organic particles. Consequently, our calculations estimate that d-BrC tar balls comprise between 5% and 26% (determined by the product of the three stated fractions) of the total PM mass concentration.

## Online content

Any methods, additional references, Nature Portfolio reporting summaries, source data, extended data, supplementary information, acknowledgements, peer review information; details of author contributions and competing interests; and statements of data and code availability are available at 10.1038/s41561-023-01237-9.

## Supplementary information

Supplementary Information Supplementary Figs. 1–7, Tables 1 and 2 and Text.

## Figures and Tables

**Extended Data Fig. 1:**
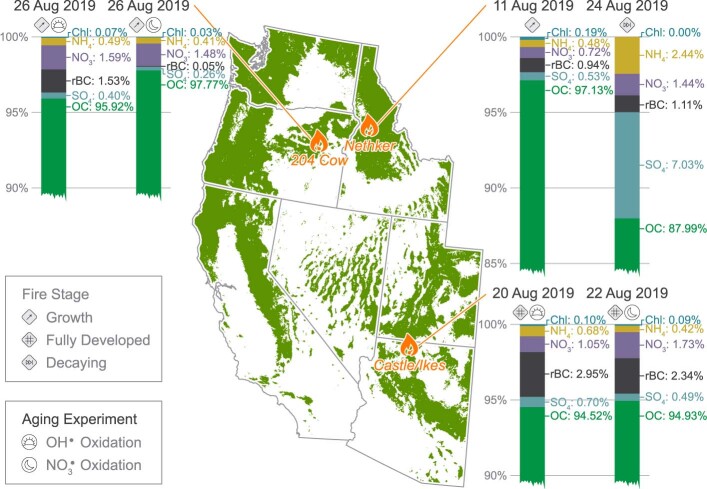
Compositional analysis of sampled smoke. A map showing the ground-based sampling location and dates of the three wildfires–Nethker/Shady Creek (Idaho), 204 Cow (Oregon), and Castle and Ikes (Arizona)–along with the fire stages investigated and any *in situ* oxidation experiment (denoted by sun and moon symbols) conducted on the intercepted smoke plumes. The colored bars show the chemical composition of smoke aerosol emissions – non-refractory matter, measured using an aerosol mass spectrometer, and refractory black carbon, measured using a single-particle soot photometer. Tree coverage data from radicalcartography.net courtesy of William Rankin.

## Data Availability

All raw data used for this study are available for public use at NASA’s FIREX-AQ data repository: https://www-air.larc.nasa.gov/cgi-bin/ArcView/firexaq. The scanning transmission electron microscopy and electron energy-loss spectroscopy data are available at https://zenodo.org/record/7402393#.Y46CuXbMJhE. The refractive index dataset is available from https://data.mendeley.com/datasets/97n9gxp5hz/1.

## Extended data

**Extended Data Table 1:** Scaling laws for dark BrC imaginary refractive index

Y	Y_0_	S	β
k_high BC fraction_	7.9 ± 2.3	λ(nm)	−0.65 ± 0.05
k_low BC fraction_	57 ± 27	λ(nm)	−1.08 ± 0.08
k_nighttime oxidation_	80 ± 180	λ(nm)	−1 ± 0.4
k_daytime oxidation_	20 ± 50	λ(nm)	−0.89 ± 0.32

Power law fitting coefficients of the form *Y* = *Y*_*0*_ *S*^*β*^ for the measured imaginary index of refraction *k* of d-BrC particles under different conditions during FIREX-AQ. Units of wavelength of light λ is in nano meters (10^−9^ meters). The interval of λ is between 350 and 1200 nm. The error bars represent one standard deviation.

## Code availability

The python code used for the analysis of the optical properties of aerosol particles is available at https://zenodo.org/record/7402393#.Y46CuXbMJhE.
