# Supplementary material for: Shortwave absorption by wildfire smoke dominated by dark brown carbon
Source: Nat Geosci. 2023 Aug 7;16(8):683–8. doi: 10.1038/s41561-023-01237-9 (PMC10409647; doi:10.1038/s41561-023-01237-9)
Supplement: Supplementary file 1 — Supplementary Figs. 1–7, Tables 1 and 2 and Text. [file 41561_2023_1237_MOESM1_ESM.pdf]

nature geoscience

Article

https://doi.org/10.1038/s41561-023-01237-9

Shortwave absorption by wildfire smoke dominated by dark brown carbon

In the format provided by the authors and unedited

Table-of-contents

Supplementary Text

Tables 1 to 2

Figs. 1 to 7

Wildfire plumes intercepted

Shady Creek Fire, Idaho: The Shady Creek fire (44.15° N, 115.019° W) was burning in the Salmon-Challis National Forest, Idaho. The fire was ignited by lightning on July 10, 2019 and burned over 6286 acres of densely forested area, which is home to eight species of conifer trees–ponderosa pine, Douglas-fir, grand fir, western larch, lodgepole pine, Englemann spruce, subalpine fir, and whitebark pine. The grass and shrub communities are dominated by Idaho fescue, bluebunch wheatgrass, stiff sagebrush, mountain big sagebrush, and bitterbrush.
Castle and Ikes Fires, Northern Arizona: The Castle and Ikes fires (36.51° N, 112.28° W, and 36.35° N, 112.29° W, respectively), were ignited by lightning on 12 and 25 July 2019, respectively, by lightning strikes. These two large fires were burning in close proximity to each other near the North Rim of the Grand Canyon, near Page, AZ. The fires burned near the Oquer Canyon in the Kaibab National Forest, a densely forested area consisting predominantly of Ponderosa pine (*Pinus ponderosa*), Douglas fir (*Pseudotsuga menziesii*), Englemann spruce (*Picea engelmannii*), and quaking aspen (*Populus tremuloides*). Minor species include Gambel oak (*Quercus gambelii*), and various shrub and grass species of sagebrush (*Artemesia*) and bitterbrush (*Purshia*). By 20 August 2019, the fire management areas had grown to approximately 24,000 acres (~97 km^2) combined.
204 Cow Fire, Eastern Oregon: The 204 Cow Fire (44.28° N, 118.47° W) took place in the Malheur National Forest region of the Blue Mountains in eastern Oregon, approximately 200 km west-southwest of McCall. The 204 Cow Fire was ignited on 9 August by lightning and grew to burn 9,668 acres (approximately 29 km^2) until its containment on 15 October. The region is predominantly forested by various species of pine, fir, and juniper trees (*Pinus, Abies,* and *Juniperus*, respectively) along with shrub species of sagebrush (*Artemesia*) (Riccardi et al. 2007). The fire had burned approximately 5,500 acres (22.3 km^2) by 26 August.

Ground-based instruments

The Aerodyne Mobile Laboratory (AML), a mobile sampling platform equipped with a suite of research-grade instrumentation, was used to sample and facilitate the *in situ* experiments. The AML travelled throughout Idaho, Oregon, Utah, and Arizona during FIREX-AQ, sampling continuously when not conducting oxidation experiments. The general sampling strategy was to search for smoke-filled valleys and transect plumes with the AML, using Tuneable Infrared Laser Direct Absorption Spectrometer (TILDAS, Aerodyne Research, Inc.) measurements of hydrogen cyanide (HCN) as a tracer for biomass smoke plumes. Upon identification of a suitable location, the AML parked with the sample inlet on the front of the truck facing into the wind to avoid self-sampling of its own exhaust. For the most part, plume sampling was conducted less than 3 km from the fire management area. The entire list of gas and particle instruments are provided in the FIREX-AQ white paper (https://www.esrl.noaa.gov/csl/projects/firex-aq/whitepaper.pdf). We provide brief description of the instruments of relevance to this project.

Multiwavelength integrated photoacoustic-nephelometer (MIPN): The MIPN is a custom-built instrument designed and constructed for ground-based operations. During FIREX_AQ, the aerosol light absorption coefficient (Mm^{-1}) was measured with the prototype version of the instrument at two wavelengths (λ = 488 and 561 nm). The photoacoustic design is based on ref[1] and the nephelometer is designed based on ref [2]. The MIPN is an improvement to the single-wavelength integrated photoacoustic-nephelometer spectrometers described in [3] and the instrument calibration procedure is detailed in ref [3] and ref [4]. The nephelometer was calibrated using NaCl aerosol and the photoacoustic spectrometer was calibrated using kerosene soot. Scattering calibration factors were determined using the slope of a linear regression of scattering versus extinction coefficient

for salt aerosol, and the absorption calibration factor was determined using the slope of absorption coefficient versus extinction-scattering coefficients for kerosene soot. The multi-wavelength beam in the MIPN is split into two identical cells wherein one cell measures the absorption and scattering coefficients of the sample stream and the other cell samples particle-free air to measure gaseous background and account for noise within the system. Measurements were acquired every 2 seconds and the readings from the clean cell were subtracted from the measurement cell to account for any gas-phase absorption. The MIPN was operated during the Castle/Ikes and 204 Cow fire ground sampling for several days.

Aerosol Mass Spectrometer (AMS): The Soot Particle-Aerosol Mass Spectrometer (SP-AMS) (Aerodyne Inc.) is a high-resolution time of flight aerosol mass spectrometer equipped with an intracavity laser vaporizer operating at 1064 nm [5]. The SP-AMS was used to measure the chemical speciation of the non-refractory as well as the refractory particles with aerodynamic diameters ranging from 70 – 2500 nm. The SP-AMS operated on a 50% duty cycle and would operate as a regular AMS when the laser was turned off. The SP-AMS was used during the Oregon experiments and the regular AMS was used for the Arizona experiments. The instrument was run with a 20 second time resolution and measured chemical speciation and mass loading by mass spectral analysis.

Potential Aerosol Mass (PAM) Reactor: A Potential Aerosol Mass (PAM, Aerodyne Research, Billerica, MA) Reactor is an oxidative flow photochemical reactor (OFR) capable of mimicking atmospheric oxidation processes using a variety of oxidants. During our experiments, the PAM was operated to generate two oxidation environments abundant in either hydroxyl (OH) or nitrate (NO_3) radicals which simulated daytime and nighttime oxidation, respectively. The PAM was equipped with two sets of 185 nm lamps where the primary set had a maximum irradiance of ~70 μW/cm^2, and the second set had a maximum irradiance of ~7 μW/cm^2. Lamp output was controlled from a software by controlling the input voltage to the lamp ballast, from 0 V (lamps off) to 10.0 V (lamps at maximum). Irradiance, relative humidity, and temperature were monitored by internal sensors. For the OH experiments, ozone (generated via the photolysis of ultra-high purity oxygen using a 185 nm lamp) was photolyzed using 185 nm lamps to produce O(^{1}d) which then reacts with water vapor to produce OH radicals[6,7]. The PAM was coupled to a laminar flow reactor (LFR) during the NO_3 experiments. The NO_3 radicals were generated in the LFR via the reaction of NO_2 with ozone to produce N_2O_5, which further reacted with ozone to produce NO_3[8].

Single-Particle Soot Photometer (SP2): The SP2 is an instrument that measures, in situ, the time-dependent scattering and incandescence signals produced by individual BC-containing particles as they travel through a continuous-wave laser beam. Any particle traversing the laser beam will scatter light, and the BC component of a BC-containing particle will absorb some of the laser energy until its temperature is raised to the point at which it incandesces (hereafter we adopt the standard terminology of the SP2 community and denote any substance determined by the SP2 to be BC as refractory black carbon (rBC)). The amplitude of the rBC incandescence signal is related to the amount of refractory material contained in the illuminated particle. By binning the individual incandescence signals per unit sample volume, the mass concentration [ng/m3] of rBC is derived. By binning the individual signals by volume equivalent diameter the size distribution (dN/dlogDVED) per unit time is derived. The rBC mass loading per unit time and the rBC size distribution unit time are the core data products produced by the SP2. Additionally, the scattering channel can be used to provide information on the rBC particle population-based mixing states

within ambient aerosols. The SP2 onboard the AML was calibrated using fullerene soot (lot L18U002) prior to the study and with lot L20W054 during the study.

Airborne Instruments

The NASA DC-8 aircraft conducted 23 individual flights during FIREX-AQ, including 13 flights characterizing wildfires in the western United States, 8 flights targeting prescribed burning plumes, and 2 transit flights. In this study we focus on the smoke sampling from the Shady Creek and Castle/Ikes wildfires by the NASA DC-8 aircraft during flights on July 25, August 12, and August 13, 2019. This is done to provide overlap with the ground based measurements of the same wildfire source.

The entire list of gas and particle instruments are provided in the FIREX-AQ white paper (https://www.esrl.noaa.gov/csl/projects/firex-aq/whitepaper.pdf). All airborne measurement datasets are archived and made open-access via https://www-air.larc.nasa.gov/missions/firex-aq/.

A three wavelength (405 nm, 532 nm, and 664 nm) photoacoustic spectrometer (PAS) was deployed on the DC-8 providing real-time measurements of dry aerosol absorption of fine particles (diameters < 2.5 µm)[9]. A cavity ringdown (CRD) aerosol extinction spectrometer composed of 8 separate ringdown cells was also deployed[10]. The CRD measures dry (<10% RH) extinction at 405, 532, and 662 nm wavelength, while two 405 nm and 532 nm cells sampled air heated to 250 ^{0}C in a thermodenuder to measure volatilize condensed coatings. Refractory black carbon (rBC) mass concentration was measured by a single particle soot photometer (SP2), which quantified rBC mass in individual particles in the 0.090 to 0.550 µm size range (volumetric-equivalent diameter assuming 1.8 g cm^{-3} void-free density) based on the incandescence signal they generated when passing through a laser beam[11]. The SP2 was calibrated using fullerene soot, lot 40971, which is a very good proxy for sensitivity to ambient rBC. Aerosol number size distribution was measured by a laser aerosol spectrometer (LAS, model 3340, TSI Incorporated, Shoreview, MN). The reported size range is from ~100 nm to 4 µm. Sampling was conducted to cover each transect of smoke, with sampling times of ~1 to 3 minutes and an airflow rate of 1.0 L/minute.

$NO_3\cdot$ experiment design

To generate $NO_3\cdot$, N_2O_5 was first generated in the gas phase from the reaction $NO_2 + O_3 \rightarrow NO_3\cdot + O_2$ followed by the reaction $NO_3\cdot + NO_2 \rightarrow N_2O_5$ in a 152.4 cm long x 2.22 cm ID perfluoroalkoxy laminar flow reactor (LFR) coupled to the OFR[8]. Separate flows containing NO_2 (1% in N_2, Praxair) and O_3 were added to the LFR. In these experiments, the NO_2 +N_2 flow rate was set between 0 and 40 cm^3 min^{-1}, and O_3 was generated by passing 1.8 L min^{-1} of O_2 through an ozone chamber housing a mercury fluorescent lamp (GPH212T5VH, Light Sources, Inc.). The O_3 mixing ratio that was input to the LFR was approximately 250 ppmv during $NO_3\cdot$-OFR experiments. The NO_2 + N_2 and O_2 flow rates were set using mass flow controllers. The N_2O_5 generated in the LFR thermally decomposed at room temperature inside the OFR to generate $NO_3\cdot$. The first oxidation step of $NO_3\cdot$-OFR experiments was with ozone only ("OFR_O3") to assess the effect of O_3 exposure on OA composition and optical properties relative to ambient OA. During $NO_{3,Arizona}$, NO_2 was stepped down from 40 to 20, 5, and 3 cm^3 min^{-1} to generate the various oxidation time scales. The integrated NO_3 exposure, defined as the product of the average $NO_3\cdot$ concentration and the mean OFR residence time (τ_{OFR}), was calculated using an estimation equation developed by Lambe et al. (2020)[8]:

$$\log\left[(\mathrm{NO_3})_{exp}\right] = a + b\log[273.15 + T_{\mathrm{OFR}}] + c\log[\tau_{\mathrm{OFR}}] + d\log[\mathrm{NO_2}]_{0,\mathrm{LFR}} + e\log[\mathrm{O_3}]_{0,\mathrm{LFR}} \times T_{OFR} + f\log\left[\mathrm{k_{w_{OFR},N_2O_5}}\right]$$

$$+\log\left(\frac{[\mathrm{NO_2}]_{0,\mathrm{LFR}}}{[\mathrm{O_3}]_{0,\mathrm{LFR}}}\right)\times\left(g\left(\log[\mathrm{O_3}]_{0,\mathrm{LFR}}\right)^2+h\log[\mathrm{O_3}]_{0,\mathrm{LFR}}\right)$$

$$-\frac{[\mathrm{NO_2}]_{0,\mathrm{LFR}}}{[\mathrm{O_3}]_{0,\mathrm{LFR}}}\times\left(i+j\log[\mathrm{O_3}]_{0,\mathrm{LFR}}\right)+k\log(\mathrm{NO_3R})_{\mathrm{ext}}$$

$$+l\log[NO_2]_{0,LFR}\times T+m\log[O_3]_{0,LFR}\times\log k_{w_{OFR},N_2O_5} \quad (1)$$

where a through m are empirical fit coefficients derived by Lambe et al. (2020)[8], T_{OFR} is the measured temperature in the OFR, $[NO_2]_{0,LFR}$ and $[O_3]_{0,LFR}$ were the NO_2 and O_3 mixing ratios input to the LFR (ppmv), $k_{wOFR,N2O5} = 0.01\ s^{-1}$ is the assumed N_2O_5 wall loss rate coefficient in the OFR, and $(NO_3R)_{ext}$ is the external NO_3· reactivity (s^{-1}), which was calculated from the summed products of ambient VOC concentrations (measured with Vocus) and their NO_3· rate coefficients (data available in the FIREX AQ data repository). Corresponding calculated NO_3· exposures was 1.4×10^{14} molec cm^{-3} s, or approximately 72 equivalent hours (3 equivalent days) of atmospheric oxidation at a 24-hour average NO_3· concentration of 5×10^{8} molec cm^{-3} based on Atkinson, 1991[12].

OH· Experiment Design

OH· was generated via photolysis of ambient O_2 and H_2O at λ = 185 nm plus photolysis of O_3 (generated from at λ = 254 nm using low-pressure mercury (Hg) lamps):

$H_2O + h\nu_{185} \rightarrow H + OH\cdot$

$H + O_2 \rightarrow HO_2$

$O_2 + h\nu_{185} \rightarrow 2O(^3P)$

$O(^3P) + O_2 \rightarrow O_3$

$O_3 + h\nu_{254} \rightarrow O_2 + O(^1D)$

$O(^1D) + H_2O \rightarrow 2OH\cdot$

A fluorescent dimming ballast was used to regulate current applied to the lamps (GPH436T5VH/4, Light Sources, Inc.). The dimming voltage applied to the ballast ranged from 1.6 V to 10 V direct current (DC). To extend the range of OH· concentrations below what is achievable with one set of lamps at 1.6 VDC, a second set of GPH436T5VH/4 lamps with added segments of opaque heat shrink tubing applied to ~86 % of the arc length[13] was used. The OH exposure was calculated using Eq. (2) from Rowe et al. (2020)[13]:

$$\log\left[\mathrm{OH_{exp}}\right]=\left(a+\left(b-c\times\mathrm{OHR}_{\mathrm{ext}}^{d}+e\times\log[\mathrm{O_3}]\times\mathrm{OHR}_{\mathrm{ext}}^{f}\right)\times\log[\mathrm{O_3}]+\log[\mathrm{H_2O}]\right)+\log\left(\frac{\tau}{124}\right) \quad (2)$$

where a through f are fit coefficients, O_3 is the ozone mixing ratio measured at the exit of the OFR (molec cm^{-3}), OHR_{ext} is the external OH· reactivity (s^{-1}), which was calculated from the summed products of ambient VOC concentrations (measured with Vocus) and their OH· rate coefficients (data available in the FIREX AQ data repository), H_2O is the ambient water vapor mixing ratio (%), and τ is the residence time in the OFR. Corresponding calculated OH· exposures was 3.91×10^{11} molec cm^{-3} s, or approximately 72 equivalent hours (3 equivalent days) of atmospheric oxidation at a 24-hour average OH· concentration of 1.5×10^{6} molec cm^{-3} per Mao et al., 2009[14].

TABLES

Table 1. Number fractions of smoke aerosol particles classified from TEM images

**Fire**	**All particle**	**Brighter particle fraction**	**Darker particle fraction**
Shady	1310	0.38	0.62
Castle/Ikes	1125	0.37	0.63
Castle/Ikes	1402	0.51	0.49

Table 2. Number fractions of soot (or BC) and dark tar balls classified from TEM images. Internally mixed soot particles were also included in the count.

**Fire**	**Dark tar balls #**	**Soot (BC) particle#**	**Dark tar ball/soot (BC)**
Shady	815	206	4.0
Castle/Ikes	709	179	4.0
Castle/Ikes	680	98	6.9

FIGURES

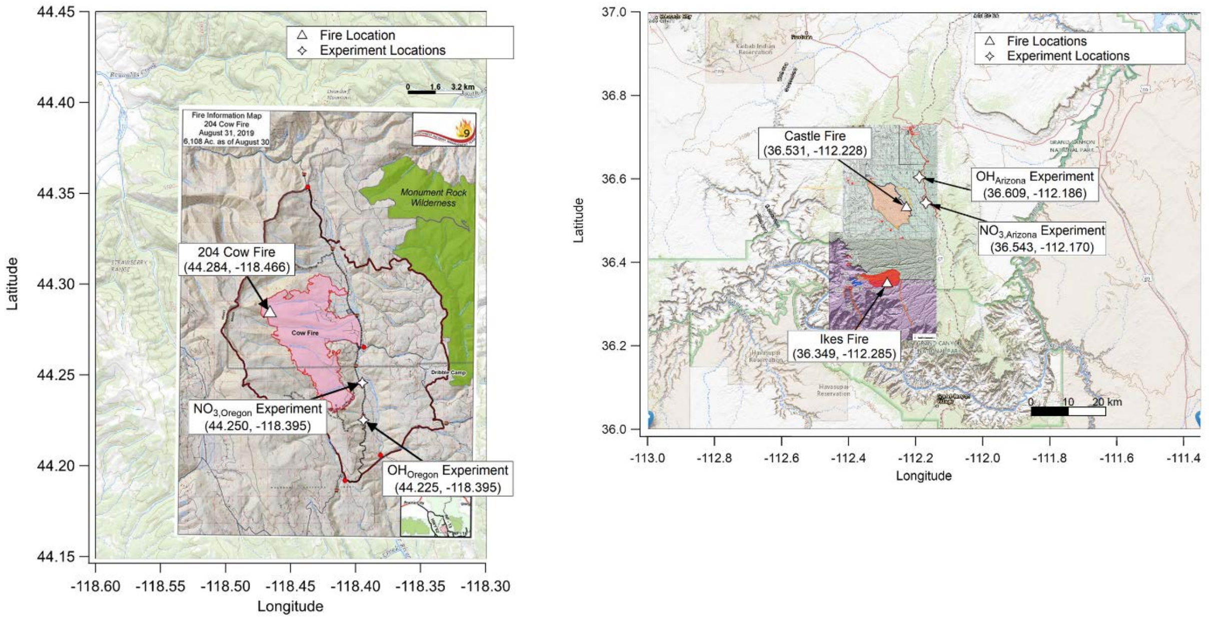

Fig 1: Maps containing the geographical coordinates of the 204 Cow Fire and Castle Fire. Fire origin locations are marked as white triangles. The oxidation experiments using the photochemical reactor are marked as white stars.

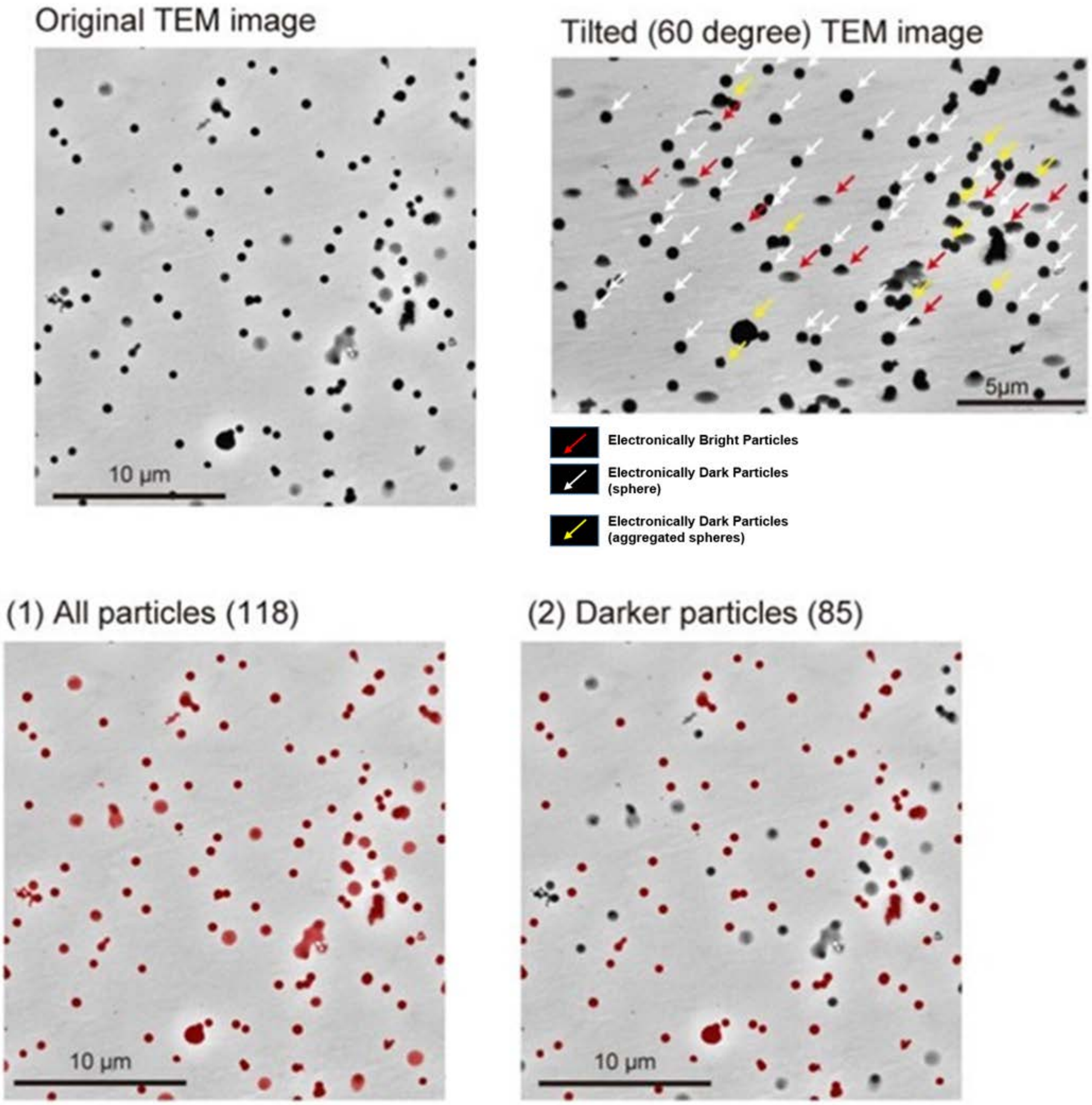

Fig 2: An example of TEM images and image processing for particles sampled on DC-8. Caution was exercised for distinguishing d-BrC tar balls in an aerosol population. The d-BrC tar balls appear as "Electronically dark" under an electron microscope[15]. Weakly absorbing or purely scattering organic matter appear as 'electronically bright'. Nearly half or more of all measured particles were classified as electronically dark particles (refer to Table 1).

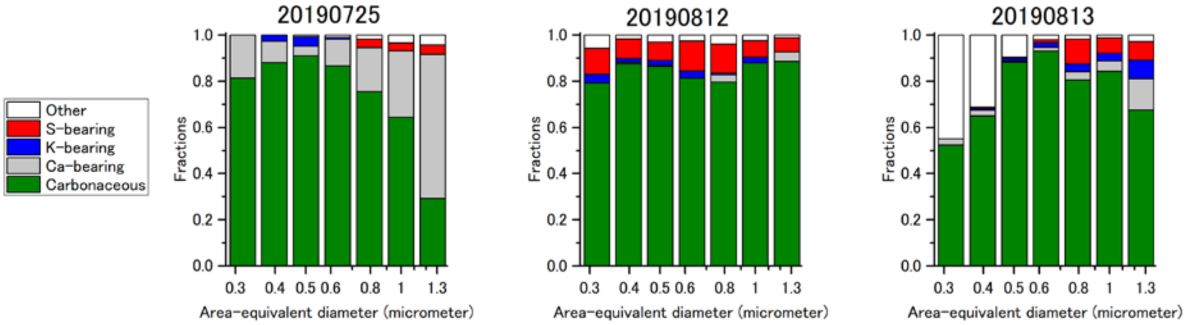

Fig 3. Size-dependent number fractions of aerosol particles in wildfire plumes from flights July 25 (Shady fire), August 12 (Castle), and August 13 (Castle). Energy dispersive X-ray spectroscopy within a TEM was used to analyze the elemental composition.

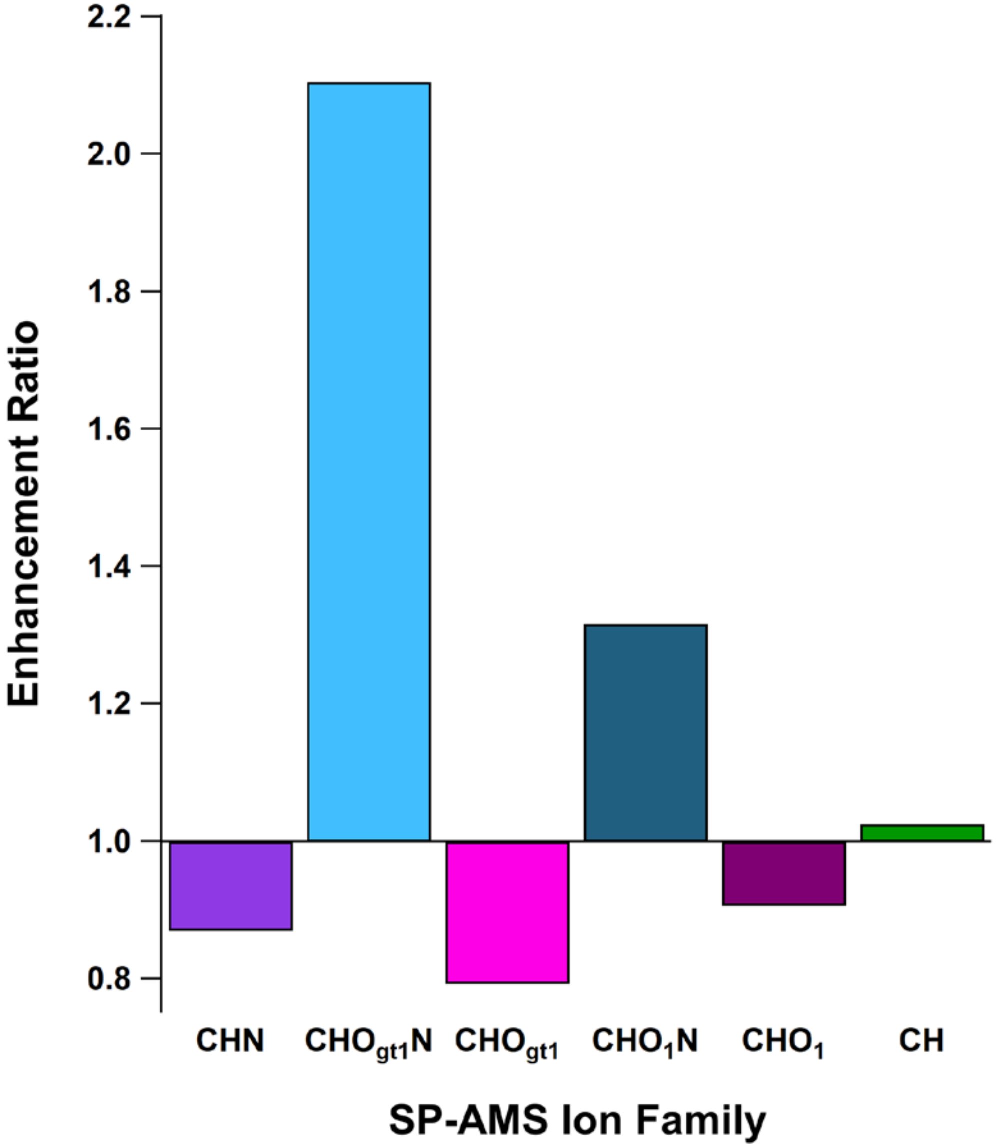

Fig. 4: Enhancement and depletion of ion families measured by the Aerodyne Mass Spectrometer (AMS) during NO3• oxidation. The $CHO_{gt1}N$ ("O_{gt1}" indicates more than one oxygen atom in a molecule containing one or more atoms of C, H, and N) family is enhanced by 211 % and CHO_1N ("O_1" indicates a single oxygen atom in a molecule containing one or more atoms of C, H, and N) by 132 % through the OFR, with a corresponding diminishment in CHO_{gt1} and CHO_1 (where, again, "O_{gt1}" and "O_1" indicate the quantity of oxygen atoms in molecules of one or more atoms of C and H) to 79 % and 91 %, respectively. This does not imply that all CHO_1N species come from CHO_1 (or $CHO_{gt1}N$ from CHO_{gt1}, similarly). The addition of nitrogen-containing functional groups is likely the cause of the observed light absorption enhancement since nitrogenated aromatic hydrocarbons form during reactions with NO_3•. This has relevance for formation of nighttime secondary organic aerosol and BrC[16].

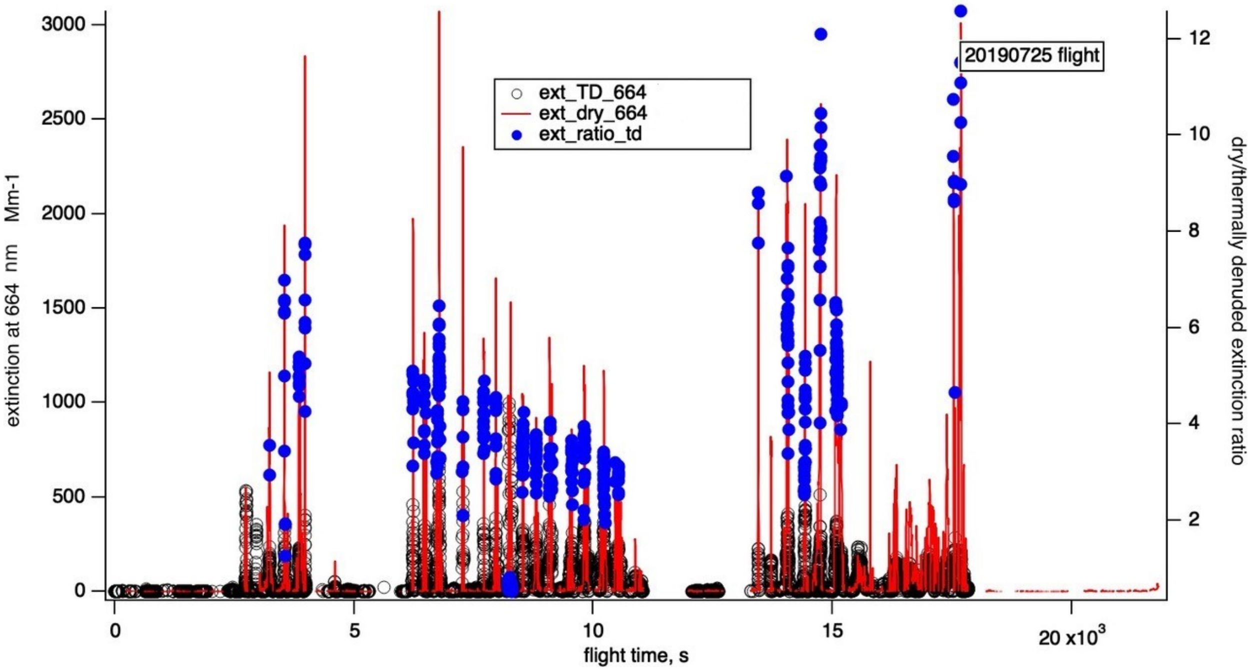

Fig. 5. Ratio of extinction at 664 nm with and without thermal denuding measured using the airborne NOAA cavity ring down instrument. The extinction measurements are shown in the red lines and the hollow black circles. Ratio of NOT-DENUDED to DENUDED is shown in blue circles, plotted against the right axis.

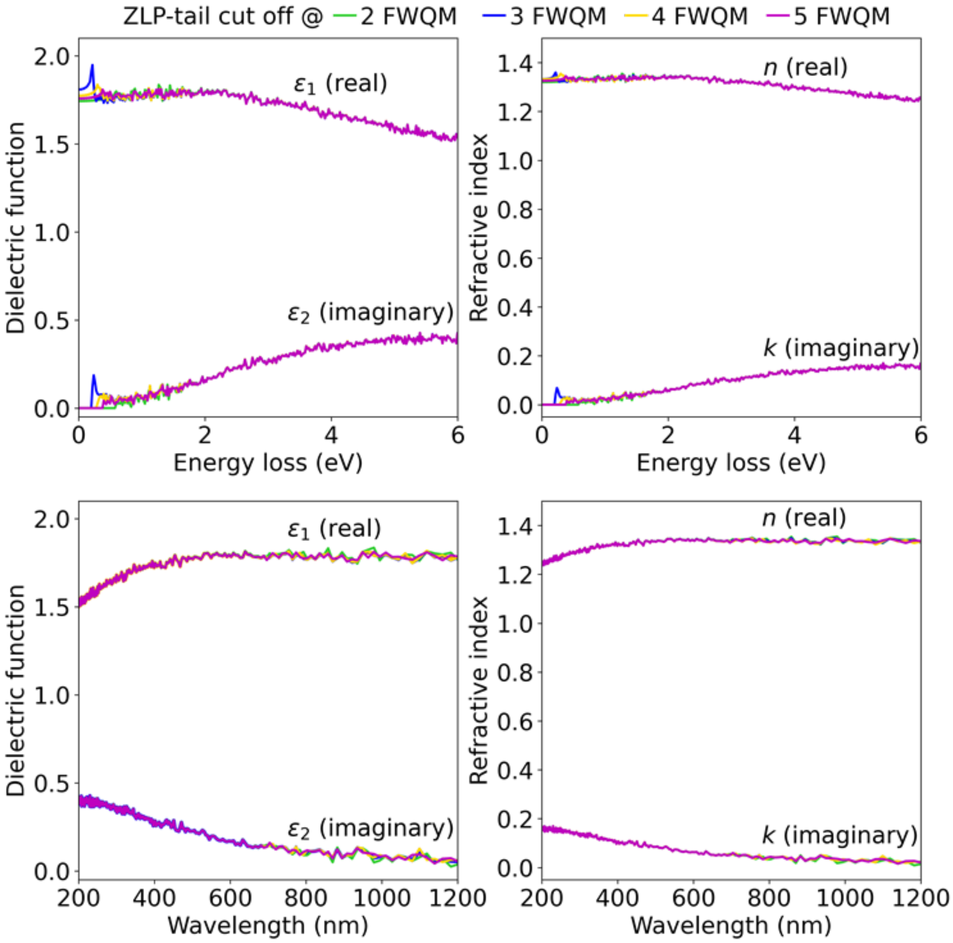

Fig 6. Variation of dielectric constants and refractive indices with the zero-loss peak (ZLP) tail cut off. Here FWQM stands for the full width at quarter maximum. The choice of the ZLP tail cutoff within the reflected tail method does not introduce any significant deviations in the dielectric constant and refractive index across the optical range.

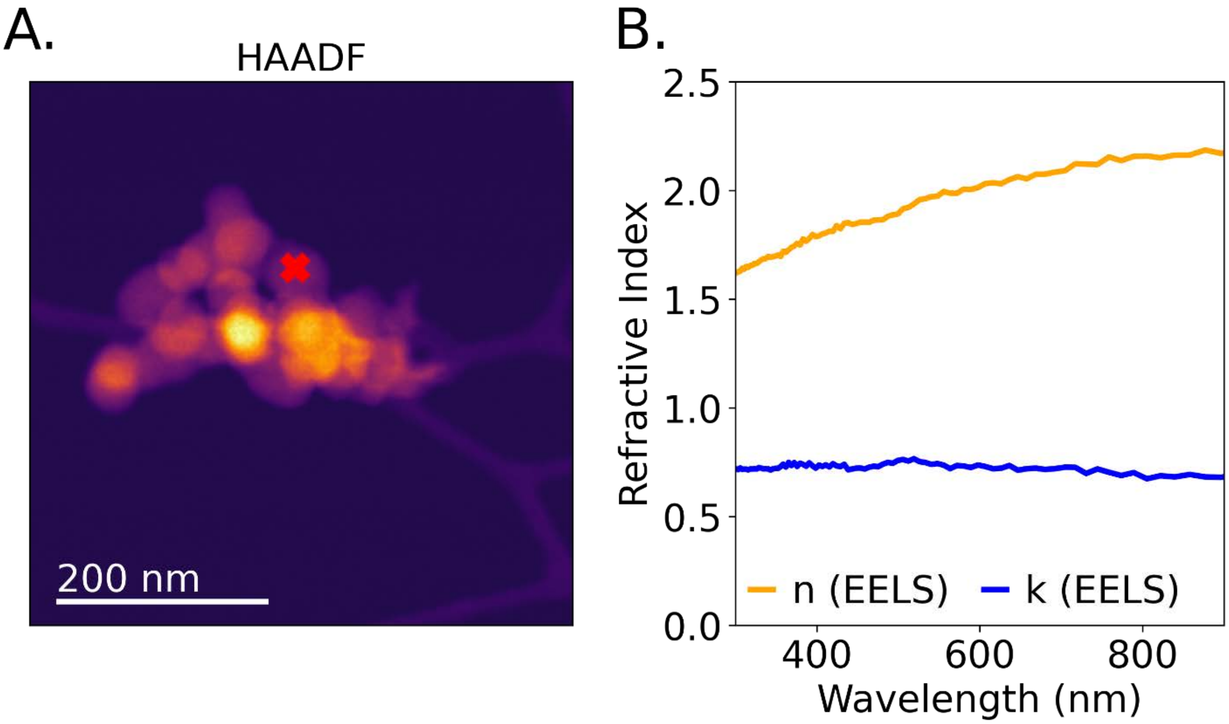

Fig. 7. (A) HAADF image showing a co-emitted BC aggregate. (B) Extracted real (*n*) and imaginary (*k*) refractive index in the optical wavelength λ range of 300-900 nm. The refractive index values were extracted from the EEL spectra acquired from the region marked as a red cross in the HAADF image. The measurement values compare well against the fixed value of 1.75 – 0.63i used for modeling the optical properties of BC across the visible spectrum in climate models[17].

References

1 Arnott, W. P., Moosmüller, H., Rogers, C. F., Jin, T. & Bruch, R. Photoacoustic spectrometer for measuring light absorption by aerosol: instrument description. *Atmospheric Environment* **33**, 2845-2852 (1999).

2 Abu-Rahmah, A., Arnott, W. & Moosmüller, H. Integrating nephelometer with a low truncation angle and an extended calibration scheme. *Measurement Science and Technology* **17**, 1723 (2006).

3 Sumlin, B. J. *et al.* Atmospheric photooxidation diminishes light absorption by primary brown carbon aerosol from biomass burning. *Environmental Science & Technology Letters* **4**, 540-545 (2017).

4 Shetty, N., Beeler, P., Paik, T., Brechtel, F. J. & Chakrabarty, R. K. Bias in Quantification of Light Absorption Enhancement of Black Carbon Aerosol Coated with Low Volatility Brown Carbon. *Aerosol Science and Technology*, 1-16 (2021).

5 Onasch, T. *et al.* Soot particle aerosol mass spectrometer: development, validation, and initial application. *Aerosol Science and Technology* **46**, 804-817 (2012).

6 Kang, E., Root, M., Toohey, D. & Brune, W. Introducing the concept of potential aerosol mass (PAM). *Atmos. Chem. Phys.* **7**, 5727-5744 (2007).

7 Lambe, A. *et al.* Characterization of aerosol photooxidation flow reactors: heterogeneous oxidation, secondary organic aerosol formation and cloud condensation nuclei activity measurements. *Atmospheric Measurement Techniques* **4**, 445-461 (2011).

8 Lambe, A. T. *et al.* Nitrate radical generation via continuous generation of dinitrogen pentoxide in a laminar flow reactor coupled to an oxidation flow reactor. *Atmospheric Measurement Techniques* **13**, 2397-2411 (2020).

9 Lack, D. A. *et al.* Brown carbon and internal mixing in biomass burning particles. *Proc. Natl. Acad. Sci. U. S. A.* **109**, 14802-14807 (2012).

10 Langridge, J. M., Richardson, M. S., Lack, D., Law, D. & Murphy, D. M. Aircraft instrument for comprehensive characterization of aerosol optical properties, Part I: Wavelength-dependent optical extinction and its relative humidity dependence measured using cavity ringdown spectroscopy. *Aerosol Sci. Technol.* **45**, 1305-1318 (2011).

11 Gao, R. *et al.* A novel method for estimating light-scattering properties of soot aerosols using a modified single-particle soot photometer. *Aerosol Sci. Technol.* **41**, 125-135 (2007).

12 Atkinson, R. Kinetics and mechanisms of the gas-phase reactions of the NO3 radical with organic compounds. *J. Phys. Chem. Ref. Data* **20**, 459-507 (1991).

13 Rowe, J. P., Lambe, A. T. & Brune, W. H. Effect of varying the λ= 185 and 254 nm photon flux ratio on radical generation in oxidation flow reactors. *Atmos. Chem. Phys.* **20**, 13417-13424 (2020).

14 Mao, J. *et al.* Airborne measurement of OH reactivity during INTEX-B. *Atmos. Chem. Phys.* **9**, 163-173 (2009).

15 China, S., Mazzoleni, C., Gorkowski, K., Aiken, A. C. & Dubey, M. K. Morphology and mixing state of individual freshly emitted wildfire carbonaceous particles. *Nature Communications* **4** (2013).

16 Decker, Z. C. *et al.* Nighttime chemical transformation in biomass burning plumes: a box model analysis initialized with aircraft observations. *Environ. Sci. Technol.* **53**, 2529-2538 (2019).

17 Brown, H. *et al.* Biomass burning aerosols in most climate models are too absorbing. *Nature communications* **12**, 1-15 (2021).
